# Assessment of bladder pressure and discomfort symptoms: How do overactive bladder differ from interstitial cystitis/bladder pain syndrome patients?

**DOI:** 10.1186/s12894-022-01164-8

**Published:** 2023-03-30

**Authors:** Angelíca Gousse, Joel Vetter, H. Henry Lai

**Affiliations:** Division of Urologic Surgery, Department of Surgery, Washington University School of Medicine, 4960 Children’s Place, Campus Box 8242-02-0022, St. Louis, MO 63110 USA

**Keywords:** Pain, Bladder sensation, Interstitial cystitis/bladder pain syndrome, Overactive bladder

## Abstract

**Background:**

To better understand the sensation of bladder “pressure” and “discomfort”, and how they are similar or distinct from the “pain” and “urgency” symptoms in IC/BPS and OAB.

**Methods:**

IC/BPS and OAB patients rated their bladder pain, pressure, discomfort, and urinary urgency on separate 0–10 numeric rating scales (NRS). Their NRS ratings were compared between IC/BPS and OAB, and Pearson correlations were performed.

**Results:**

Among IC/BPS patients (n = 27), their mean numeric ratings of pain, pressure, discomfort, and urinary urgency were almost identical (6.6 ± 2.1, 6.0 ± 2.5, 6.5 ± 2.2, and 6.0 ± 2.8 respectively). The three-way correlations between pain, pressure, or discomfort were very strong (all > 0.77). Among OAB patients (n = 51), their mean numeric ratings of pain, pressure, and discomfort (2.0 ± 2.6, 3.4 ± 2.9, 3.4 ± 2.9) were significantly lower than urgency (6.1 ± 2.6, *p* < 0.001). The correlations between urgency and pain, and between urgency and pressure were weak in OAB (0.21 and 0.26). The correlation between urgency and discomfort was moderate in OAB (0.45). The most bothersome symptom of IC/BPS was bladder/pubic pain, while the most bothersome symptom of OAB was urinary urgency and daytime frequency.

**Conclusions:**

IC/BPS patients interpreted bladder pain, pressure, or discomfort as the similar concepts and rated their intensity similarly. It is unclear whether pressure or discomfort provide additional information beyond pain in IC/BPS. Discomfort may also be confused with urgency in OAB. We should re-examine the descriptors pressure or discomfort in the IC/BPS case definition.

**Supplementary Information:**

The online version contains supplementary material available at 10.1186/s12894-022-01164-8.

## Background

The descriptors “pain, pressure or discomfort” are used collectively to describe the abnormal bladder or pelvic sensations associated with interstitial cystitis/bladder pain syndrome (IC/BPS). For example, the AUA/SUFU Guideline defines IC/BPS as “an unpleasant sensation (pain, pressure, discomfort) perceived to be related to the urinary bladder, associated with lower urinary tract symptoms […]” [1]. The ESSIC case definition of IC/BPS describes “chronic (> 6 months of) pelvic pain, pressure, or discomfort perceived to be related to the urinary bladder accompanied by at least one other urinary symptoms such as persistent urge to void or frequency […]” [2]. Although the reference to bladder pain in IC/BPS is universally accepted, the reason why “pressure” or “discomfort” are also included in the descriptors of IC/BPS is not entirely clear.

Presumably “pressure” or “discomfort” were included along with “pain” because not all IC/BPS patients complained of pain. Humphrey et al. noted that some IC/BPS patients mentioned bladder discomfort and pressure in addition to bladder pain during qualitative interviews [3]. Bogart et al. performed a systematic review of symptom prevalence of IC/BPS and showed that although pain distinguished IC/BPS from overactive bladder (OAB), some patients characterized their pain as pressure, aching, or burning [4]. Despite these studies, it is still not clear how often IC/BPS patients complain of “pressure” or “discomfort” without pain; and how severe the sensation of bladder “pressure” or “discomfort” compared to pain. It is also not known whether the descriptors “pressure” or “discomfort” provide additional or differentially useful information beyond the pain descriptor in IC/BPS, or help to distinguish IC/BPS from other confusable conditions such as OAB. To our knowledge, bladder “pressure” or “discomfort” have not been systematically studied in IC/BPS patients.

Overactive bladder (OAB) syndrome is defined by the cardinal symptom of urinary urgency [5]. However in qualitative interview studies, an additional theme emerged as some OAB patients described their urgency as “pressure” or “discomfort” [6]. For example, one illustrative quote from an OAB patient said: “I think the *pressure* is the *urgency*. When I get the *pressure* I’ve gotta make a dash for the bathroom because it is very *uncomfortable*.” [6] About 30% of OAB patients reported that their urge to urinate was mainly because of “pain, pressure, or discomfort” [7]. This can lead to confusion since “pressure or discomfort” are also embedded in the definitions of IC/BPS [1, 2]. In our clinical experience, some OAB patients described their abnormal bladder sensation in terms of pressure or discomfort, in addition to the classic sudden urgency to urinate.

In this study, we asked IC/BPS and OAB patients to rate their bladder “pain”, “pressure”, “discomfort”, and urinary “urgency” sensation on separate numeric rating scales. We then compared their mean ratings and correlations, to discover group differences between IC/BPS and OAB patients. Our objective is to better understand the sensation of bladder “pressure” and “discomfort”, and how they are similar or distinct from “pain” (the cardinal symptom of IC/BPS) and “urgency” (the cardinal symptom of OAB) in the two clinical populations. This study may provide further clarity on the distinguishing factors between bladder pain, pressure, discomfort and urgency symptoms.

## Materials and methods

### Population

Patients who presented to urology clinic and were diagnosed clinically with IC/BPS or OAB by one urologist (HHL) consented to participate in this questionnaire study between late 2012 and early 2014 (the timing of the enrollment was related to funding). The clinical diagnosis of IC/BPS and OAB was consistent with the AUA IC/BPS Guideline [1] and the 2002 ICS definition [5] respectively, and generally taken into accounts all clinical factors including chief complaint, history, physical examination findings, and laboratory and diagnostic workup as indicated. IC/BPS patients had an unpleasant sensation (pain, pressure, discomfort) perceived to be related to the bladder, associated with lower urinary tract symptoms of more than 6 weeks duration, in the absence of infection or other identifiable causes. OAB patients had urinary urgency, with or without urgency urinary incontinence, usually with frequency and nocturia, and in the absence of infection or other identifiable causes. Consistent with the AUA Guidelines, cystoscopy and urodynamics were not performed on most of the patients, so we had no further information on detrusor overactivity for OAB, or Hunner lesion status for IC/BPS.

### Assessment

Patients completed the following questions to rate their bladder pain, pressure, discomfort, and urinary urgency on separate 0–10 numeric rating scales (NRS):

1. Think about the pain associated with your bladder. On average, how would you rate these symptoms in the past 4 weeks? (0 = no pain, 10 = most severe pain I can imagine)
2. Think about the pressure associated with your bladder. On average, how would you rate these symptoms in the past 4 weeks? (0 = no pressure, 10 = most severe pressure I can imagine)
3. Think about the discomfort associated with your bladder. On average, how would you rate these symptoms in the past 4 weeks? (0 = no discomfort, 10 = most severe discomfort I can imagine)
4. Think about the urgency to urinate associated with your bladder. On average, how would you rate these symptoms in the past 4 weeks? (0 = no urgency, 10 = most severe urgency I can imagine)

We intentionally chose not to define these descriptors for the patients because we wanted the patients to interpret their bladder sensations for themselves when rating them.

In addition, patients were also asked to identify their single most bothersome symptom over the past 4 weeks among the followings: (1) pain, pressure, discomfort in the pubic or bladder area, (2) pain, pressure, discomfort in the area between your perineum (male) or vaginal area (female), (3) pain, discomfort during or after sexual activity, (4) strong need to urinate with little or no warning, (5) frequent urination during the day, (6) frequent urination at night, (7) sense of not emptying your bladder completely, (8) other. For the most bothersome symptom question, we did not separate out pain from pressure or discomfort. The exact wordings of these questions were identical to the SYM-Q questionnaire administrated in the MAPP Study.

As mentioned earlier, the clinical diagnosis of IC/BPS and OAB was based on patient chief complaint, history, physical examination findings, and laboratory and diagnostic workup as indicated in accordance to the AUA Guidelines on IC/BPS and OAB [1, 5]. The clinical assignment to IC/BPS and OAB were not based on patients’ response to the 0–10 NRS questions here. The responses to the 0–10 NRS questions were not reviewed until data analysis a few years after the patients were originally seen and diagnosed.

Additional questionnaires used to characterize the cohorts included: (1) genitourinary pain index (GUPI) [8], (2) interstitial cystitis symptom and problem indexes (ICSI, ICPI) [9], (3) international consultation on incontinence modular questionnaire-overactive bladder (ICIQ-OAB) [10], (4) international consultation on incontinence modular questionnaire-urinary incontinence short form (ICIQ-UI) [11], (5) Overactive Bladder Questionnaire Short Form (OAB-q) [12]. All participants signed an informed consent. The IRB approved this study, #IRB 201208077.

### Statistics

For comparisons between IC/BPS and OAB cohorts, Wilcoxon sum-rank tests and chi-square tests were used to compare continuous and categorical data respectively. Paired t-tests were used to compare within group variables of interest. Spearman’s rank coefficient was used to assess correlation between quantitative variables. All analyses were performed using R version 4.1.0 with statistical significance set at *p* < 0.05. Since formal comparison of pain, pressure, and discomfort ratings have never been done in these two populations, a priori power calculation was not performed.

## Results

### Characteristics and demographics of the cohorts

There were no statistical differences in age, sex, and race between the IC/BPS and OAB cohorts. Both men and women were represented, and the cohorts were predominantly white (see Additional file 1: Table S1). As expected, IC/BPS participants had significantly higher ICSI, ICPI and GUPI-Pain Subscale scores (*p* = 0.003, < 0.001, 0.002, respectively), while OAB participants had significantly higher scores on OAB-q, ICIQ-UI, and ICIQ-OAB (*p* < 0.001, 0.002, 0.007, respectively).

### Comparison of ratings of pain, pressure, discomfort, and urgency

Among IC/BPS patients (n = 27), their mean numeric ratings of pain, pressure, discomfort, and urinary urgency were almost identical (6.6 ± 2.1, 6.0 ± 2.5, 6.5 ± 2.2, and 6.0 ± 2.8 respectively, pairwise *p* all > 0.05), see Table 1.

**Table 1:** NRS of pain, pressure, discomfort, and urgency in IC/BPS and OAB

Variable	IC/BPS	OAB	*p* value
NRS (0–10)	(n = 27)	(n = 51)	
Bladder pain			< 0.001
Mean	6.6	2.0	
SD	2.1	2.6	
Bladder pressure			< 0.001
Mean	6.0	3.4	
SD	2.5	2.9	
Bladder discomfort			< 0.001
Mean	6.5	3.4	
SD	2.2	2.9	
Urinary urgency			0.949
Mean	6.0	6.1	
SD	2.8	2.6	

Among OAB patients (n = 51), their mean numeric ratings of pain, pressure, and discomfort (2.0 ± 2.6, 3.4 ± 2.9, 3.4 ± 2.9) were significantly lower than urgency (6.1 ± 2.6, *p* all < 0.001). There was a significantly bigger difference in ratings between bladder pain and urgency (urgency minus pain = 4.1 ± 3.2) than between bladder pressure and urgency (urgency minus pressure = 2.8 ± 3.3, *p* < 0.001) and between bladder discomfort and urgency (urgency minus discomfort = 2.7 ± 2.9, *p* < 0.001) among OAB patients.

### Correlations between pain, pressure, discomfort, and urgency

**Fig. 1:**
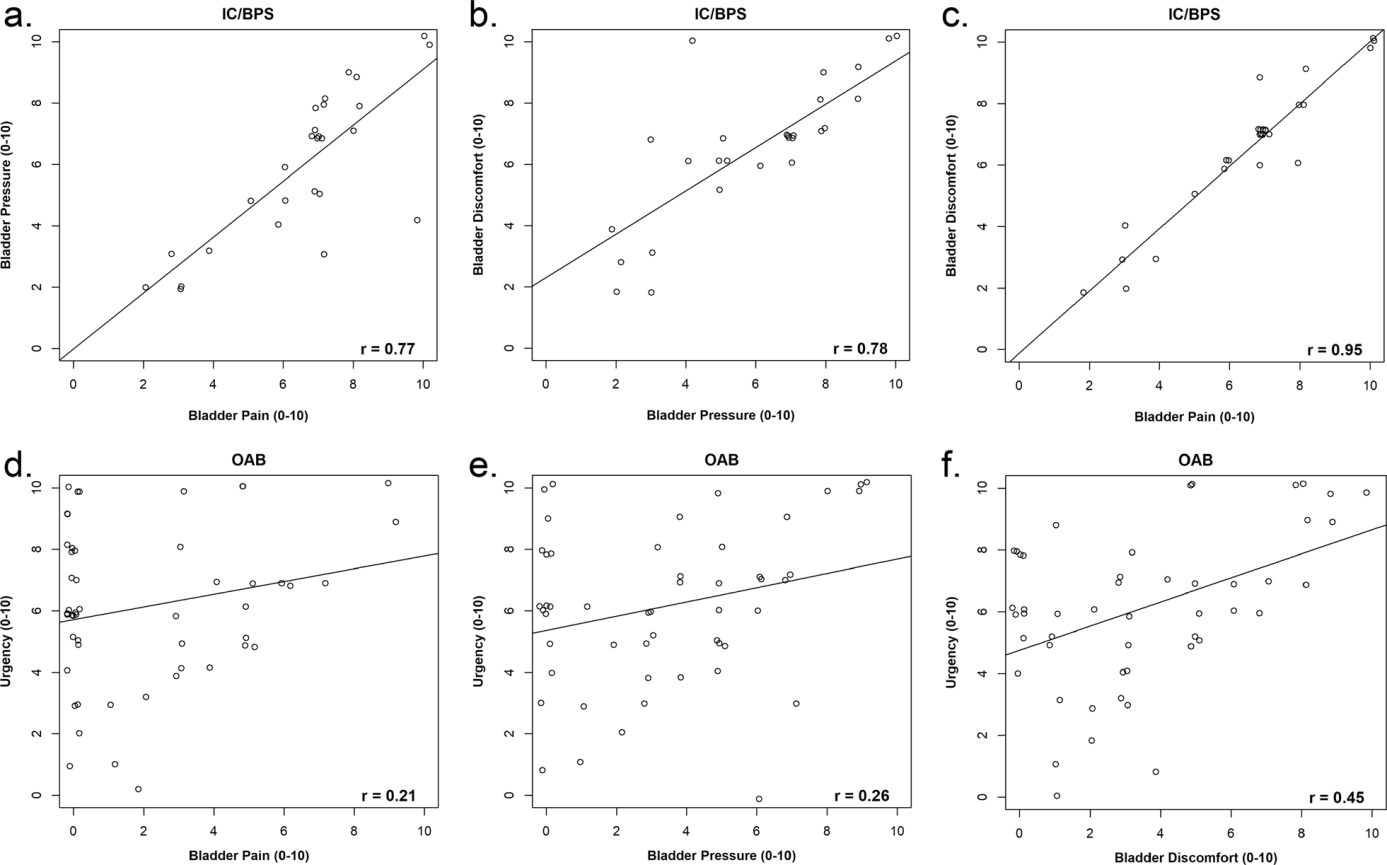
Scatterplots of **a** pain versus pressure in IC/BPS, **b** pressure versus discomfort in IC/BPS, **c** pain versus discomfort in IC/BPS, **d** urgency versus pain in OAB, **e** urgency versus pressure in OAB, **f** urgency versus discomfort in OAB. R values reported in figures

Among IC/BPS patients, the three-way correlations between pain, pressure, or discomfort were very strong (r all > 0.77), see Table 2. The highest correlation was between bladder pain and discomfort (r = 0.95) which was nearly a straight line, see Fig. 1c. The scatterplots are shown in Fig. 1a–c. Given the similar ratings between pain, pressure, discomfort, and urgency, the correlations between pain, pressure, or discomfort to urgency were also quite high among IC/BPS (0.69 to 0.80).

**Table 2:** Pearson correlation coefficients between pain, pressure, discomfort, and urgency for (a) IC/BPS, (b) OAB

	Pain	Pressure	Discomfort	Urgency
(a) IC/BPS—Pearson correlation coefficient table				
Pain	–	0.774	0.949	0.692
Pressure	–	–	0.780	0.799
Discomfort	–	–	–	0.708
Urgency	–	–	–	–
(b) OAB—Pearson correlation coefficient table				
Pain	–	0.633	0.612	0.212
Pressure	–	–	0.530	0.261
Discomfort	–	–	–	0.452
Urgency	–	–	–	–

Among OAB patients, the correlations between pain and urgency (the cardinal symptom of OAB), and between pressure and urgency were weak (0.21 and 0.26). The correlation between discomfort and urgency was moderate in OAB (r = 0.45). The scatterplots are shown in Fig. 1d–f.

### Most bothersome symptom

The most bothersome symptom for over half of IC/BPS patients (51.9%) was pain, pressure or discomfort localized in the bladder and pubic region rather than the perineal area in men or vaginal area in women (7.4%). While a small percentage of OAB patients (9.8%) did complain of pain in the bladder and pubic area (and none in the perineal or vaginal area), the most bothersome symptoms were urinary urgency and daytime frequency (41.2% for both). Approximately 41.2% of OAB patients reported urgency as their most bothersome symptom in contrast to ~ 4% in IC/BPS patients (*p* < 0.001). Frequency during the day was identified as the most bothersome symptom in 41.2% of OAB patients and 18.5% in IC/BPS patients (*p* < 0.05). In addition, 25.5% of OAB patients reported urinary frequency at night as the most bothersome symptom.

The comparisons between IC/BPS and OAB patients were shown in Table 3.

**Table 3:** Self-reported most bothersome symptoms among IC/BPS and OAB patients

Most bothersome symptom % (N)*	IC/BPS (n = 27)	OAB (n = 51)	*p* value
Pain/pressure/discomfort in bladder/pubic area	51.9% (14)	9.8% (5)	< 0.001
Pain/pressure/discomfort in perineum/vaginal area	7.4% (2)	0.0% (0)	0.117
Pain/discomfort after sexual activity	11.1% (3)	5.9% (3)	0.412
Urgency	3.7% (1)	41.2% (21)	< 0.001
Frequency during the day	18.5% (5)	41.2% (21)	0.048
Frequency at night	14.8% (4)	25.5% (13)	0.390
Sense of not emptying bladder	3.7% (1)	21.6% (11)	0.049
Other	11.1% (3)	0.0% (0)	0.038

*The % may add up to over 100% since some patients marked more than one response

### The presence of any non-zero (NRS > 0) pain, pressure, discomfort, or urgency

One question we asked earlier was: how often did IC/BPS patients report pressure or discomfort without pain? In our IC/BPS cohort, there was no patient who reported any pressure or discomfort without pain. Every single patient reported pain, pressure, AND discomfort. 26 of 27 (96.3%) IC/BPS patients also reported non-zero urinary urgency or frequency along with pain.

Among OAB patients, 50 of 51 (98%) reported non-zero urinary urgency, 24 (47.1%) reported non-zero bladder pain, 36 (70.6%) reported non-zero pressure, and 39 (76.5%) reported non-zero discomfort.

## Discussion

In this study, we compared the bladder pain, pressure, discomfort, and urgency sensation between IC/BPS and OAB patients, in order to better understand how bladder “pressure” and “discomfort” are similar or distinct from “pain” (the cardinal symptom of IC/BPS) and “urgency” (the cardinal symptom of OAB) in the two clinical populations.

In our cohort of IC/BPS patients, there was no patient who reported any pressure or discomfort without pain. Every single patient reported pain, pressure, AND discomfort. Thus we were unable to compare IC/BPS patients with pressure or discomfort alone versus those with pain. Their mean numeric ratings of pain, pressure, or discomfort were nearly identical. In addition, the three-way correlations between pain, pressure, or discomfort were very strong (r all > 0.77). In fact, the correlation between pain and discomfort ratings was extremely high (r = 0.95), almost approaching a straight line (Fig. 1c). This suggests that IC/BPS patients interpreted pain, pressure, and discomfort as the similar concepts or generalized category of painful noxious sensation without a strong distinction between “pain”, “pressure” versus “discomfort”. The concurrent use of three descriptors “pain”, “pressure”, or “discomfort” to define IC/BPS appeared to be redundant in our small cohort of IC/BPS patients. It is unclear from our data whether the descriptors “pressure” or “discomfort” provide additional or differentially useful information beyond the pain descriptor in our small IC/BPS cohort.

Among OAB patients, the correlations between urgency and pain, and between urgency and pressure were very weak (0.21 and 0.26 respectively). The correlation between urgency and discomfort was moderate in OAB (r = 0.45). This suggest that OAB patients can distinguish urinary urgency from pain and pressure, but this distinction is less clear cut between urgency and discomfort symptoms. It is conceivable that “urgency” may be confused with “discomfort” by some OAB patients, or at least the distinction between these terms may vary among patients. This observation is consistent with a qualitative study which showed that OAB patients described their urgency as “pressure” or “discomfort [6]. If bladder “discomfort” is included in the definition of IC/BPS, some OAB patients may potentially be misclassified as having IC/BPS. The benefit of including “pressure” or “discomfort” in the definition of IC/BPS is unclear from our data. “Pressure” or “discomfort” do not discriminate between IC/BPS and OAB. It may actually add to the confusion for some OAB patients. Considering these results collectively, and upon further validation of our findings, we should consider re-examining the descriptors “pressure” or “discomfort” in the IC/BPS case definition.

Pain, on the other hand, is a much better descriptor distinguishing IC/BPS from OAB. Even though a subset of OAB patients reported non-zero bladder pain, their NRS of pain was mild (2.0 ± 2.6 out of 10) and was significantly lower than those reported by IC/BPS. More importantly, it was their most bothersome symptom that distinguished OAB from IC/BPS. The most bothersome symptom of IC/BPS patients is pain in the bladder/pubic area. The most bothersome symptoms of OAB patients are urinary urgency and frequency. These findings clearly confirmed “pain” and “urgency” as cardinal and most bothersome symptom of IC/BPS and OAB, respectively.

The report of non-zero (albert mild) bladder pain in OAB might raise the questions whether we could have misdiagnosed IC/BPS for OAB, and vice versa. However, this possibility was low since 100% of our IC/BPS patients reported pain while 98% of OAB our patients reported urgency. The distribution of their single most bothersome symptoms also aligned well with the accepted case definitions of IC/BPS and OAB. Based on the current evidence, it is possible that the subgroup of OAB patients with pain may have different underlying pathophysiology (e.g., central sensitization) from OAB patients without pain [13–16]. This hypothesis needs further investigation.

Potential limitations of the study included the small sample size, and that our cohort was predominantly white in the United States. It is possible that patients in other ethnic groups, cultures, or in other countries might interpret these bladder sensations differently from our cohort. Future studies with larger sample size and a more diverse demographics or ethnic makeup should be conducted to further examine our findings.

In future studies, it would also be beneficial to examine whether the discomfort was related to “fear of pain” (which will point to IC/BPS) versus “fear of leakage” (which will point to OAB).

## Conclusions

IC/BPS patients interpreted bladder pain, pressure, or discomfort as the similar concepts and rated their intensity similarly. It is unclear whether the descriptors pressure or discomfort provide additional information beyond the pain descriptor in IC/BPS. Bladder discomfort may also be confused with urgency in OAB. We should re-examine the descriptors pressure or discomfort in the IC/BPS case definition.

## Supplementary Information

**Additional file 1. Supplemental Table 1**. Characteristics and demographics.

## Data Availability

Study data are available upon request and pertinent to mutual agreement of sharing and using data. The datasets used or analyzed during the current study are available from the corresponding author on reasonable request.

## Abbreviations

IC/BPS: Interstitial cystitis/ bladder pain syndrome
OAB: Overactive bladder
NRS: Numeric rating scale
