# Supplementary material for: Assessment of bladder pressure and discomfort symptoms: How do overactive bladder differ from interstitial cystitis/bladder pain syndrome patients?
Source: BMC Urol. 2023 Mar 30;23:53. doi: 10.1186/s12894-022-01164-8 (PMC10061968; doi:10.1186/s12894-022-01164-8)
Supplement: Supplementary file 1 — Additional file 1. Supplemental Table 1. Characteristics and demographics. [file 12894_2022_1164_MOESM1_ESM.docx]

## Supplemental Table 1: Characteristics and Demographics

|  | | | IC/BPS | | OAB | *p*-value (adjusted for age and sex) |
| --- | --- | --- | --- | --- | --- | --- |
| *Demographics:* |  |  |  |  |  |  |
| No. of subjects | | | 27 | | 51 |  |
| Age (mean ± SD) | | | 54.2 ± 12.3 | | 53.8 ± 11.9 | 0.984 |
| Sex (% female) | | | 56.7% | | 72.5% | 0.143 |
| Race (% white) | | | 63.3% | | 43.1% | 0.079 |
| *UI/OAB measures (mean ± SD):* | | | |  |  |  |
| ICIQ-UI (urinary incontinence, 0-21) | | | 1.4 ± 2.0 | | 12.0 ± 4.9 | 0.002 |
| ICIQ-OAB (overactive bladder, 0-16) | | | 2.0 ± 1.5 | | 9.3 ± 2.6 | 0.007 |
| OAB-q symptom bother (6-36) | | | 2.2 ± 2.8 | | 18.7 ± 6.7 | <0.001 |
| *IC/BPS measures (mean ± SD):* | |  |  |  |  |  |
| ICSI (0-20) | | | 1.9 ± 2.9 | | 30.2 ± 16.6 | 0.003 |
| ICPI (0-16) | | | 0.9 ± 1.4 | | 12.7 ± 5.6 | <0.001 |
| GUPI-Pain Subscale (0-23) | | | 0.1 ± 0.4 | | 8.8 ± 8.2 | 0.002 |

The comparison between the population characteristics and demographics of IC/BPS and OAB patients were shown in Supplemental Table1. There were no statistical differences in age, sex, and race between the IC/BPS and OAB cohorts. Both men and women were represented, and the cohorts were predominantly white.
